# Microfluidic chip enables single-cell measurement for multidrug resistance in triple-negative breast cancer cells

**DOI:** 10.20517/cdr.2019.77

**Published:** 2020-03-11

**Authors:** Karan Parekh, Hamideh Sharifi Noghabi, Jose Alejandro Lopez, Paul Chi Hang Li

**Affiliations:** ^1^Department of Molecular Biology & Biochemistry, Simon Fraser University, Burnaby V5A 1S6, Canada.; ^2^Department of Chemistry, Simon Fraser University, Burnaby V5A 1S6, Canada.; ^3^School of Environment & Science, Griffith University, Nathan, Queensland 4111, Australia.

**Keywords:** MDA-MB-231 cell, triple-negative breast cancer, microfluidic chip, lab-on-chip, multidrug resistance, single-cell analysis, fluorescence measurement

## Abstract

**Aims:** Triple-negative breast cancer patients are commonly treated with combination chemotherapy. Nonetheless, outcomes remain substandard with relapses being of a frequent occurrence. Among the several mechanisms that result in treatment failure, multidrug resistance, which is mediated by ATP-binding cassette proteins, is the most common. Regardless of the substantial studies conducted on the heterogeneity of cancer types, only a few assays can distinguish the variability in multidrug resistance activity between individual cells. We aim to develop a single-cell assay to study this.

**Methods:** This experiment utilized a microfluidic chip to measure the drug accumulation in single breast cancer cells in order to understand the inhibition of drug efflux properties.

**Results:** Selection of single cells, loading of drugs, and fluorescence measurement for intracellular drug accumulation were all conducted on a microfluidic chip. As a result, measurements of the accumulation of chemotherapeutic drugs (e.g., daunorubicin and paclitaxel) in single cells in the presence and absence of cyclosporine A were conducted. Parameters such as initial drug accumulation, signal saturation time, and fold-increase of drug with and without the presence cyclosporine A were also tested.

**Conclusion:** The results display that drug accumulation in a single-cell greatly enhanced over its same-cell control because of inhibition by cyclosporine A. Furthermore, this experiment may provide a platform for future liquid biopsy studies to characterize the multidrug resistance activity at a single-cell level.

## Introduction

Despite the recent progression in cancer treatments, breast cancer is still the most common female malignancy worldwide^[1]^. In breast cancer, malignant epithelial cells can be unresponsive or hyperactive to the hormones, estrogen, and progesterone^[2]^, resulting in uncontrolled cellular growth and proliferation. Triple-negative breast cancer (TNBC), which lacks expression of the estrogen, progesterone, and HER2 receptor, is often a basal-like breast cancer capable of growing high-grade aggressive tumors that can spread quickly. As a result, neither hormonal therapy nor targeted therapy is offered for triple-negative or basal-like breast cancers^[2]^. Therefore, many cancer patients are prescribed with conventional chemotherapies and targeted radiation therapies, which generally involve a combination of drugs and regimens. While novel immunotherapies have shown great promise in several cancers, in TNBC, only very tentative initial trials have been completed. It appears that the best option for those approaches to be effective will be as part of a mix of therapeutic approaches that also includes chemotherapy and targeted therapies.

A major drawback to the success of combination chemotherapy in TNBC patients is the non-responsive nature of cancer cells to a range of chemotherapeutic agents, an occurrence known as multidrug resistance^[2,3]^. A major cause of multidrug resistance (MDR) involves the hyperactive efflux of hydrophobic cytotoxic drugs mediated by ATP-binding cassette (ABC) proteins in mammary epithelial cells. Three commonly observed ABC proteins, which are relevant in MDR in breast cancer, include ABC transporter subfamily B member 1 or P-glycoprotein, ABCC1, and breast cancer resistance protein (or ABC subfamily G member 2)^[4-8]^. Such protein transporters function as efflux pumps for hydrophobic compounds, which intensify the transport of chemotherapeutic drugs, resulting in an ultimate reduction of the intracellular drug concentration and ineffective cancer therapy. Inhibition of these ABC proteins can possibly inverse the cells’ MDR mechanism and cultivate a prospect to increase the cellular drug accumulation, with the intention of improving the success of cancer therapy^[9]^. The use of ABC transporter inhibitors such as cyclosporine A^[10]^ and fumitremorgin C^[11]^ have been previously reported.

Traditionally, MDR function was studied by measuring drug accumulation using a variety of methods including, but not limited to, radiolabeling-based microtiter plate assays and flow-cytometry^[12]^. However, such methods have been publicized to be mechanically demanding and time-consuming with additional drawbacks. First, these methods are “bulk assays” and thus such experiments require cell samples in large numbers to generate statistically significant results; however, obtaining cells in great number is not always possible. Moreover, biological heterogeneity is notable only at an individual cell level, and such large cell populations are heterogeneous and therefore bulk assays cannot reveal the MDR variations at the cellular level^[13]^.

To address these issues, we adopted the microfluidic chip technology, which has greatly advanced in past few decades, enabling researchers to study many disease mechanisms^[14-18]^. The microfluidic chip has been simulated, designed, and tested for studying polymeric particles and biological cells^[19-23]^. Moreover, in comparison with conventional methods, the microfluidic method has many advantages when using liquid biopsy samples^[24]^. Firstly, because of the narrow size of the microchannel, it is easier to isolate a single cell in the microchannel than in a bulk solution. Second, because of no evaporation of cell medium, microfluidic experiments on a single-cell level can be performed for an extended period of time without the concern of cell death. Lastly, no change in fluorescence will occur owing to the variation in the liquid depth at the observation region. As a result, single-cell assays can retain details of individual cells that are otherwise masked out in bulk cell assays^[19,20,23]^.

In this paper, the MDR assay of drug accumulation was performed in the absence of, and then presence of, an MDR inhibitor to permit the same cancer cell to serve as its own internal control. In addition, we demonstrate that the single-cell method provides time-dependent cellular drug transport data and permits morphological interrogation, which allows us to categorize different single cells.

## Methods

### Microfluidic chips

**Figure 1:**
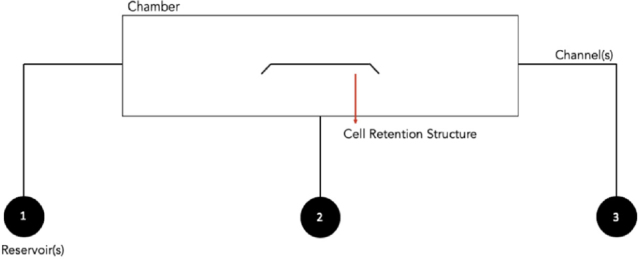
Design layout of the microfluidic chip. A structure of the chip, with the left (1) and right (3) reservoirs serving as the cell inlet and waste reservoirs, respectively, whereas the middle reservoir (2) is used for reagent delivery

The microfluidic chip (30 mm × 30 mm), as shown in Figure 1, comprises three major reservoirs, three channels, and a single chamber which contains a cellular retention structure. The microfluidic chip was fabricated by the standard micromachining processes at CMC Microsystems (Kingston, ON, Canada), which included standard glass cleaning, photolithography, photoresist development, reservoir forming on a cover plate, and chip bonding^[25,26]^. Reservoirs 1 and 3 (Left and Right) function as cell inlet and waste reservoirs, respectively, whereas Reservoir 2 channels drug delivery. Each channel is 40 μm deep, whereas the reservoirs, which are 2.5 mm in diameter, are 0.6 μm deep.

### Fluorescence detection system

**Figure 2:**
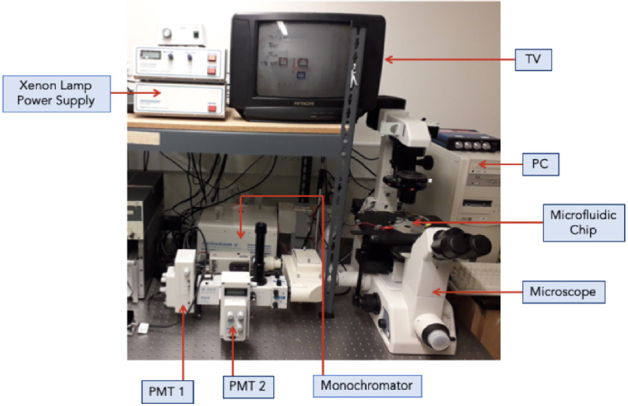
Nikon-PTI optical measurement-imaging system setup. PMT: photomultiplier tube

In Figure 2, an optical imaging system, consisting of an inverted microscope (TE300, Nikon, Mississauga, ON, Canada), was connected to a video camera (TK-CC3180; JVC, Yokosuka, Japan) for simultaneous fluorescence measurements and bright-field imaging, as previously described^[27-29]^. A TV (Hitachi) and VCR system (JVC) was used for easy microscopic observation. A video capture card (ATI-TV Wonder bt878, Markham, ON, Canada) was installed in the computer for image capture.

Optical excitation (470 nm) was provided by a Xenon arc lamp (Photon Technologies International, London, Ontario, Canada) coupled to a monochromator. A dichroic filter (620 nm) was used for only red light to enter the video camera for cell imaging, whereas the green fluorescent emission (535 nm) was transmitted to the microphotometer system (PTI, Charlotte, NC) through a detection aperture. Data were processed by the Felix Software (PTI).

### Cell samples and reagents

For the MDR assays, daunorubicin (DNR, Sigma-Aldrich, St. Louis, MO) and cyclosporine A (CsA, Sigma-Aldrich) were dissolved in dimethyl sulfoxide (DMSO, Sigma-Aldrich) to make stock solutions of 350 μM and 1 mM, respectively. Working solutions of 35-μM DNR and 5-μM and 10-μM CsA were prepared by diluting stock solutions in Hanks’ Balanced Salt Solution (HBSS, Invitrogen Corp., Grand Island, NY). Working solutions of 1-μM Oregon Green-labeled paclitaxel (OG-PTX) and 10-μM CsA were prepared by diluting stock solutions in a similar fashion. Post-assay cell viability was tested using 0.4% trypan blue solution (ThermoFisher Scientific, Waltham, MA).

The MDA-MB-231 breast cancer cell lines were obtained from cryopreserved storage. The cells were maintained in Hyclone Dulbecco’s Modified Eagle Medium (DMEM)/High Glucose (GE Life Science, Marlborough, MA) with 10% fetal bovine serum (FBS, Life Technologies, Grand Island, NY) and 1% penicillin (Stemcell Technologies, Vancouver, BC) at 5% CO_2_ and 37 °C. The doubling time for the cell lines was ~38 h; hence, growth medium was changed three times a week and cells were passaged every 72 h.

### Selection and retention of single cells

**Figure 3:**
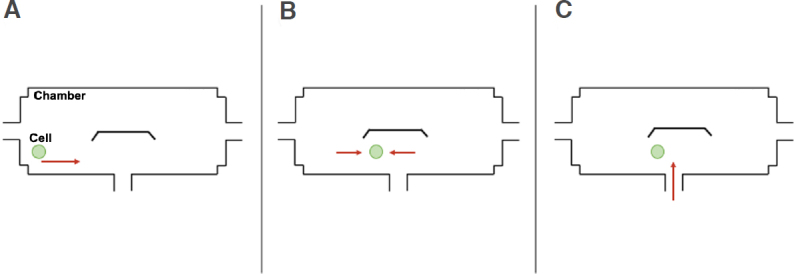
Schematic diagrams displaying how a single breast cancer cell was sorted and retained near the cell retention structure. A: Cell suspension was inserted through Reservoir 1; B: The liquid level of Reservoirs 1 and 3 was adjusted to make the cell stop near the cell retention structure; C: Upon cell retention, chemical reagents were administrated through Reservoir 2

Mammary epithelial cells are adherent and therefore will readily adhere to the surface of the microchip, as reported in previous microchip studies with adherent cells^[29,30]^. Prior to starting the experiment, the reservoirs and channels of the microfluidic chip were washed using a soap solution, rinsed with double distilled water, and sterilized with 75% ethanol. Once the chip was washed and sterilized, a 5-μL aliquot of cell medium solution was added to Reservoirs 2 and 3 to craft an environment suitable for cell survival. Thereafter, a 5-μL aliquot of cell suspension was introduced into the cell inlet (Reservoir 1; see Figure 1 for notations), and a small amount of cell medium was removed from Reservoir 3, resulting in the cell moving from left to right, as shown in the schematic diagram for the transport and retention of the breast cancer single-cell in Figure 3. When the cell moved close to the cell retention structure, some cell medium was added to the right (Reservoir 3). In this way, the liquid pressure difference was established, and a desired cancer cell was moved and stabilized in position near or around the cell retention structure. Single cells were selected based on size and morphological criteria for drug accumulation measurements.

Once the cell remained stationary in position, a 10-min incubation period was provided for the cell to adhere to the surface of the microchip. After the cell had adhered to the chamber surface, liquid from all three reservoirs was removed and fresh medium solution was added to all three reservoirs. The cell was then settled for an additional 5 min before fluorescence measurements commenced.

### Drug accumulation measurement on the single cancer cells

**Figure 4:**
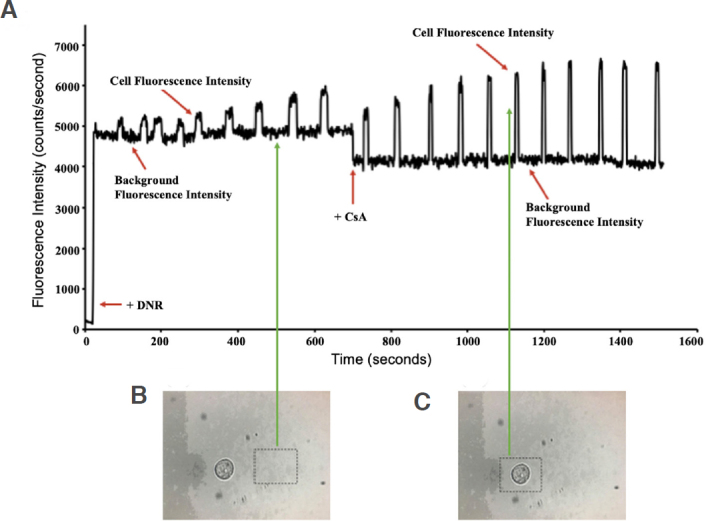
A: The raw experimental fluorescence data are plotted against time with added DNR (35 μM) at 50 s. After ~700 s, DNR (35 μM) + CsA (5 μM) was added. The fluorescence intensity was greatly enhanced, reaching its maximum peak due to non-competitive inhibition of the ABC transporters by CsA; B: Fluorescence measurement of detection window (dotted black box) are marked to show the location to measure the background signals; C: Fluorescence measurement was conducted within the detection window (dotted black box) to monitor the cell signals. DNR: daunorubicin; CsA: cyclosporine A; ABC: ATP binding cassette

After a single MDA-MB-231 breast cancer cell had adhered to the chamber of the microfluidic chip and been selected for, based on size and morphological criteria, the observation aperture for fluorescence measurement was adjusted, and two detection boxes, which equated to a size approximately 1 cm larger than the cell, were marked on the TV monitor to provide a visual observation during the experiment. Afterward, cell media in all reservoirs were removed, and then the drug inlet reservoir was filled with a 5-μL aliquot of 35-μM DNR. Fluorescence measurement (l_ex_ = 470 nm; l_em_ = 585 nm)^[24,30,31]^ began, which was to monitor the increase in fluorescence due to the accumulation of DNR in the cell in the absence of CsA, an MDR inhibitor. Thereafter, DNR accumulation was measured in the same single cell in the presence of a 5-μL aliquot of 5-μM CsA to observe an enhanced drug accumulation due to MDR inhibition. Similar experiments were run using 1-μM OG-PTX in the presence and absence of 10-μM CsA. The cell signal and the background were measured at two different detection sites, within the cell and outside the cell. To achieve this, the position of the chip was controlled by manually adjusting the chip back and forth between two detection sites. In Figure 4B, the detection site was outside the cell, in which the extracellular fluorescence intensity was detected. Figure 4C, in which the detection site was on the cell, provides the total fluorescence intensity. Translation of the chip was conducted in such a way that the aperture measured cellular fluorescence for 10 s and extracellular fluorescence for 60 s. Subtraction of the cell signal from the background gave a corrected signal representing the drug accumulation fluorescence in the cell. After all experiments, cells were treated with trypan blue, a small negatively charged dye, which only stains cells with a compromised cell membrane in order to test cell viability^[32]^.

## Results

Initially, a single TNBC cell was introduced and retained in the microfluidic chip. Then, fluorescence measurement of the retained cell was conducted using the fluorescence detection system for ~1600 s. The cancer cell was first treated with 35-μM DNR for ~700 s, as shown in Figure 4A. Fluorescence signals of the cell were shown to have increased relative to the background, noted at time points (s) 100, 160, 200, 240, 300, 360, 440, 530, and 610, averaging at 578 ± 108 counts/s. However, the signals were not high because of the efflux-mediated nature of the ABC transporters in the absence of an MDR inhibitor.

Subsequently, the cell was treated with 5-mM CsA together with 35-mM DNR for ~800 s. The results show that, after the cell was treated with DNR in the presence of CsA, an MDR inhibitor, it inhibited the drug efflux process, resulting in MDR reversal, and thus increasing the drug accumulation within the single cell. The fluorescence intensity of the cell began to increase around 890 s and enhanced dramatically afterwards, averaging at 2109 + 406 counts/s. This was caused by the diffusion of DNR into the cell being unaffected regardless of CsA. However, regardless of the uptake of DNR, the ABC transporters were inhibited by CsA. Background-corrected data were obtained by subtracting the background fluorescence from the total fluorescent intensity, and the signal from the cell that represented the dye accumulation in the single cell was given. Background correction from the raw data and fold-increase values were obtained by utilizing the following described formulas:

Equation 1:

[Fluorescence Intensity]_DNR_ = [Fluorescence]_CELL + BACKGROUND_ - [Fluorescence]_BACKGROUND_

Equation 2:

Fold-Increase of DNR = Fluorescence Signal of Inhibitor Blocked Cell/Fluorescence Signal of Unblocked Cell

**Figure 5:**
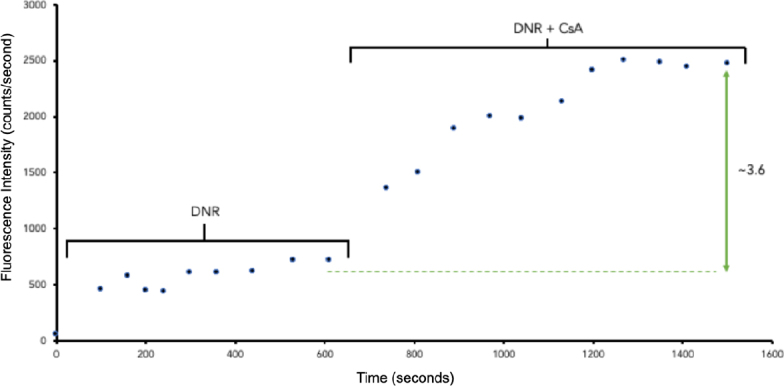
The background corrected experimental fluorescence data (counts/s) due to daunorubicin accumulation in the cell is plotted against time (s). DNR: daunorubicin; CsA: cyclosporine A

Based on the background-corrected data, it was shown that the addition of CsA caused the accumulation of DNR to increase by 3.6 folds, as shown in Figure 5. The experiment of DNR was repeated with both 5-μM and 10-μM CsA, and the data are listed in Table 1. The low fold increase was likely caused by the cell being obtained from a higher cell passage, which might suggest a loss in MDR ability.

**Table 1:** The average background-corrected fluorescence data (counts/s) for both DNR and OG-PTX (drug only and drug + inhibitor)

Name of drug	Drug only	Drug + 5 µM CsA	Drug + 10 µM CsA
DNR (35 µM)	824 ± 203	1543 ± 201	2527 ± 275
	578 ± 108	2109 ± 406	NA
OG-PTX (1 µM)	-	804 ± 95	2335 ± 130
	836 ± 269	NA	2097 ± 267

DNR: daunorubicin; CsA: cyclosporine A; OG-PTX: Oregon Green-labeled paclitaxel; NA: not available

**Figure 6:**
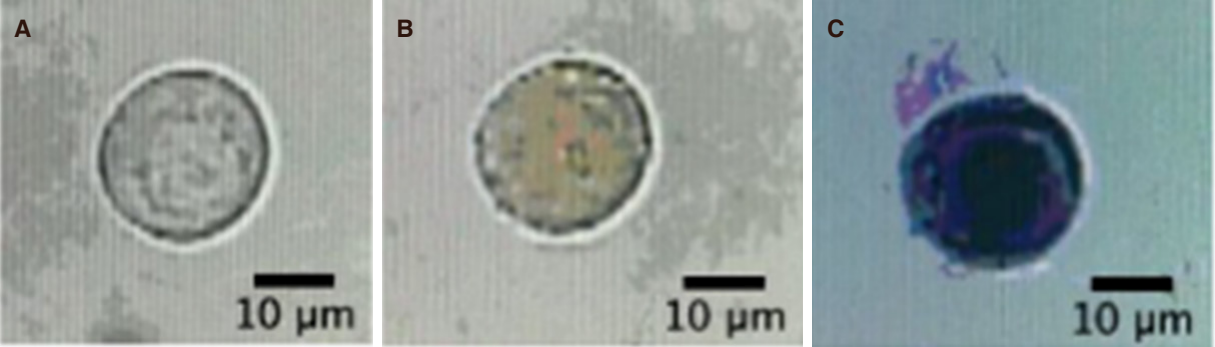
MDR Assay for a single MDA-MB-231 cell. A: Image of the singe cell prior to MDR assay; B: Image of the cell after the MDR assay; C: Image of the cell after the addition of trypan blue, with a 5-min incubation time. MDR: multidrug resistance

Figure 6 shows the images of the captured MDA-MB-231 cell before and after the experiment, and after treatment with trypan blue. According to the first image, the cell was round and in a good morphology before the experiment. After treatment with DNR and CsA, the cell’s shape changed slightly and appeared orange in regions of the nucleus, indicating the diffusion of DNR into the cell. When trypan blue was used to treat the cell as a final step, it was stained dark blue due to dye penetration through the porous cell membrane into the cytoplasm, thus indicating cell death.

**Figure 7:**
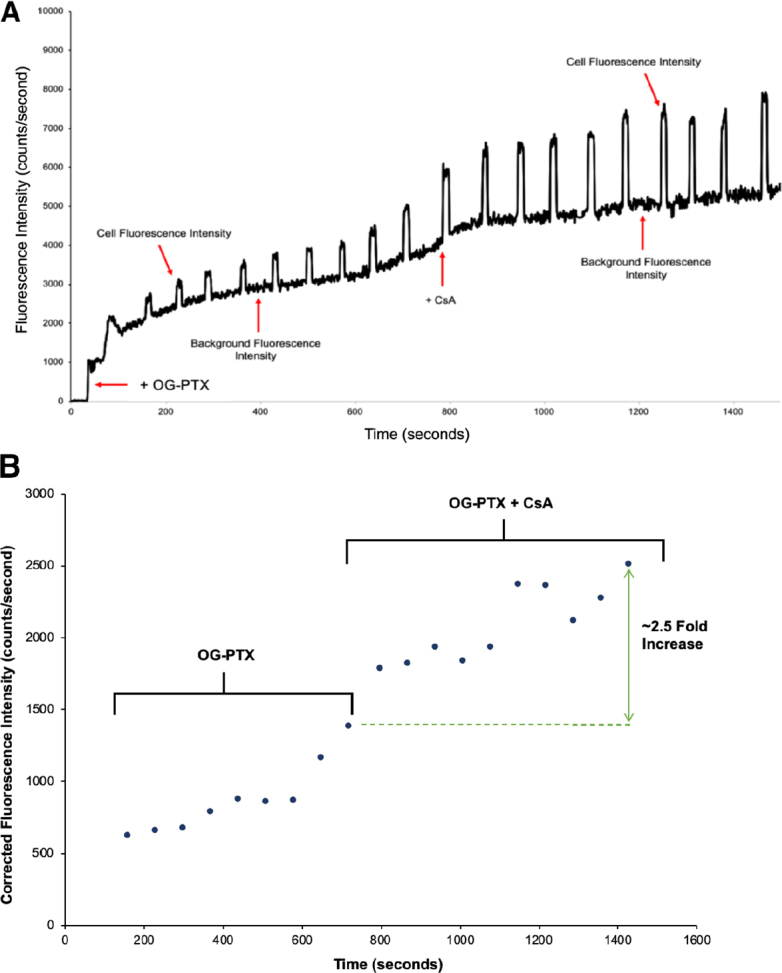
A: The raw experimental fluorescence data are plotted against time with added OG-PTX (1 μM) at 50 s. After ~800 s, OG-PTX (1 μM) + CsA (10 μM) was added. The fluorescence intensity was enhanced, reaching its maximum value; B: The background corrected experimental fluorescence data (counts/s) due to OG-PTX accumulation in the cell are plotted against time (s). CsA: cyclosporine A; OG-PTX: Oregon Green-labeled paclitaxel

A similar experiment was run using a labeled drug, OG-PTX, and the fluorescent data are shown in Figure 7. As shown in Figure 7A, a breast cancer cell was first treated with 1-μM OG-PTX for ~800 s. The results show that, after the cell was treated with the drug in the presence of CsA, it inhibited the drug efflux process, thus increasing the accumulation of OG-PTX within the single cell. As shown in the background corrected graph in Figure 7B, the fluorescence intensity of the cell began to increase around 900 s and enhanced dramatically afterward, averaging at 2097 + 267 counts/s, as listed in Table 1. Based on the background-corrected data, it was shown that the addition of CsA caused the accumulation of OG-PTX to increase by ~2.5 folds. The experiment of OG-PTX was repeated, and the data are listed in Table 1. The enhancement of fluorescence intensity of the drug from 5-μM to 10-μM of CsA was apparent.

## Discussion

This paper reports a microfluidic chip to measure the drug accumulation in single breast cancer cells in order to understand the inhibition of drug efflux properties. Single-cell selection, loading of drugs, and fluorescence measurement for intracellular accumulation of drugs were all conducted on a microfluidic chip. As a result, measurements of the accumulation of chemotherapeutic drugs (e.g., daunorubicin and paclitaxel) in single cells in the presence and absence of CsA were conducted. The results show that drug accumulation in a single-cell greatly enhanced over its same-cell control due to the inhibition by cyclosporine A. This measurement method is limited to one single cell at a time; however, this limitation can be partly resolved by incorporating multiple cell retention structures in the same single cell footprint. This work may provide a platform for future studies to characterize the MDR activity of single cells obtained in liquid biopsy samples.

## Declarations

### Availability of data and materials

The raw data is available upon email request to PCH Li.

### Copyright

© The Author(s) 2020.
